# Future care preparation profiles and self-support ability among rural Chinese older adults: a cross-sectional study

**DOI:** 10.3389/fpubh.2026.1919757

**Published:** 2026-09-03

**Authors:** Fangyuan Wang, Xianhong Li, Jiajia Han, Yinghui Guan, Jiaqi Tian, Le Xu, Bing Huang, Jianhui Xie

**Affiliations:** 1 Xiangya School of Nursing, Central South University, Changsha, China; 2 Department of Orthopedics, The Affiliated Children’s Hospital of Xiangya School of Medicine, Central South University (Hunan Children’s Hospital), Changsha, Hunan, China; 3 Hospital Administration Office, The Affiliated Children’s Hospital of Xiangya School of Medicine, Central South University (Hunan Children’s Hospital), Changsha, Hunan, China

**Keywords:** latent profile analysis, preparation for future care, rural older adults, self-efficacy, self-support ability

## Abstract

**Background:**

Population aging is accelerating worldwide, and rural older adults may face particular challenges because of limited access to formal care resources. Preparation for future care (PFC) is a multidimensional process involving awareness, avoidance, decision-making, and concrete planning. This study identified latent PFC profiles among rural older adults in China and examined their association with self-support ability.

**Methods:**

A cross-sectional survey was conducted among 851 older adults from 16 rural villages in Hebei Province, China. Data were obtained using a general information questionnaire, the 14-item Preparation for Future Care Needs Scale, the Self-supporting Ability Rating Scale for the Elderly, and the Generalized Self-Efficacy Scale. Latent profile analysis used the four PFC dimension mean scores as continuous indicators. Independent-samples *t* tests or one-way analysis of variance were used for univariate comparisons, followed by multiple linear regression.

**Results:**

Three PFC profiles were identified: scarce-preparation (23.97%), moderate-preparation (47.00%), and high-avoidance/high-action (29.03%). In the adjusted model, marital status, family members’ health status, self-efficacy, and PFC profile membership were significantly associated with self-support ability. Compared with the high-avoidance/high-action profile, the scarce-preparation profile (*B* = −52.874, *p* < 0.001) and moderate-preparation profile (*B* = −31.296, *p* < 0.001) had lower self-support ability scores. Self-efficacy was positively associated with self-support ability (*B* = 2.519, *p* < 0.001). The model explained 64.0% of the variance in self-support ability.

**Conclusion:**

PFC among rural older adults is heterogeneous, and high avoidance can coexist with active care preparation. The action-oriented components of preparation may be particularly relevant to later-life autonomy. Profile-informed, family-sensitive, and community-based approaches warrant further evaluation in longitudinal and intervention studies.

## Background

Population aging is a major and rapidly growing public health challenge worldwide. The World Health Organization estimates that by 2030 one in six people globally will be aged 60 years or older, and the global population aged 60 years and older is projected to reach 2.1 billion by 2050 (1). China is already experiencing population aging on a large scale: by the end of 2024, 310.31 million people were aged 60 years or older, accounting for 22.0% of the population (2). This demographic shift is increasing demand for long-term care and supportive services (3). Rural older adults may be particularly vulnerable because they often have fewer accessible care resources and more limited service support, while their self-support ability is closely related to available social support (4). In this context, preparation for future care (PFC) provides an important framework for understanding how older adults anticipate possible care needs and plan for later life (5). Although interest in PFC has increased, evidence from Chinese rural communities remains limited (6).

PFC is a multidimensional construct rather than a single care-related behavior, making person-centered methods useful for identifying heterogeneous preparation patterns (7). Sörensen et al. conceptualized future care preparation as attitudes and actions related to anticipating and planning for later-life care (8). The Chinese adaptation of the Preparation for Future Care Needs Scale supports the cultural applicability of this construct among Chinese older adults (9). Self-support ability, which incorporates economic self-reliance, daily self-care, and health self-maintenance, reflects an older person’s capacity to maintain autonomy in later life (10). Self-efficacy may also influence this capacity because it reflects confidence in managing challenges and implementing intended actions (11).

Latent profile analysis (LPA) has been used in nursing and behavioral research to identify subgroups within complex psychosocial and health-related constructs (12). In a closely related study of rural Chinese older adults, Li et al. identified four PFC profiles, including scarce-preparation, high-avoidance/low-action, moderate-preparation, and high-avoidance/high-action patterns (13). These findings show that avoidance and active preparation are not necessarily opposite ends of a single continuum.

Person-centered latent-variable approaches have also been proposed for other care-related needs among Chinese older adults. Xu et al. published a sequential exploratory mixed-methods protocol focused on community-based rehabilitation (CBR) needs in Guizhou Province, including questionnaire development, a pilot validation study, a large cross-sectional survey, and latent class analysis (14). Although both studies use person-centered latent-variable approaches to examine heterogeneity among older Chinese adults, they are independent studies. The earlier protocol focuses on CBR needs in Guizhou, whereas the present study focuses on PFC and self-support ability among rural older adults in Hebei. They are therefore complementary rather than sequential studies.

Expectations regarding future care may also be shaped by intergenerational support, long-term care arrangements, and structural differences in health service utilization (15, 16). In rural China, later-life care is often negotiated among spouses, adult children, and community resources, suggesting that PFC should be understood within local care systems rather than as an entirely individual choice (17). Self-care disability is common among older adults in China (18), disability trajectories in later life are heterogeneous (19), and person-centered research has identified subgroup differences in self-support ability among rural older adults (20). Qualitative work further shows that care in rural communities is embedded in family and community networks (21, 22).

The context of future care preparation is also changing as rural older adults increasingly encounter technology-enabled health services and remote monitoring (23, 24). Family functioning remains important because communication and role distribution can influence whether future care needs are discussed and planned (25). Evidence from rural Japan suggests that everyday self-management is associated with quality of life in later life (26), while social capital and mutual assistance may provide additional external support for later-life care (27). However, the relationship between PFC profile membership and self-support ability among rural older adults remains insufficiently examined. Therefore, this study aimed to (1) identify latent profiles of PFC among rural older adults using LPA and (2) examine the association between PFC profile membership and self-support ability after accounting for relevant demographic characteristics and self-efficacy.

## Methods

### Study design and setting

A cross-sectional study was conducted in 16 rural villages in Hebei Province, China. Five study sites were located in the Shijiazhuang area (Xiwangzhuang, Sangjiazhuang, Xiwan, Yujia, and Xiaopei villages), and 11 were located in Dingzhou (Dongliuchun, Shaocun, Beishaocun, Xiwangnou, Mafucai, Xichaili, Lujiazhuang, Wujiazhuang, Majiazhuang, Liuliangzhuang, and Wudingzhuang villages). Detailed administrative locations are provided in Supplementary Table S1. Rural older adults were recruited using convenience sampling. Before participation, eligible individuals were informed of the study purpose, content, and procedures, and written informed consent was obtained. The study was approved by the Medical Ethics Committee of Hebei General Hospital (approval No. 2026-LW-024).

### Participants

The inclusion criteria were: (1) age 60 years or older; (2) registered rural household status; (3) continuous residence in the local area for at least 3 years; (4) ability to communicate and understand the questionnaire and to complete it independently or with assistance from a trained investigator when necessary; and (5) voluntary participation with written informed consent. Exclusion criteria were long-term residence in a nursing institution and severe cognitive impairment or psychiatric disorders that prevented questionnaire completion. Based on the planned LPA and allowing for invalid responses, the target sample size was at least 800 participants. A total of 1,000 questionnaires were distributed, and 851 valid questionnaires were retained, yielding an overall effective response rate of 85.10%. Site-specific distribution counts were not retained in the anonymized analytic dataset and therefore site-specific response rates could not be reliably reconstructed.

### Measures

The present analysis used data from the general information questionnaire, the Preparation for Future Care Needs Scale, the Self-supporting Ability Rating Scale for the Elderly, and the Generalized Self-Efficacy Scale. The general information questionnaire included gender, age, marital status, educational level, religious belief, living arrangement, care from children, family members’ health status, number of chronic diseases, monthly household income per capita, and access to medical services.

Preparation for future care was assessed using the Chinese 14-item Preparation for Future Care Needs Scale (9). The scale comprises four dimensions: avoidance of care planning (3 items), concrete planning (4 items), awareness of future care needs (4 items), and decision-making about care preferences (3 items). Items are rated from 1 (strongly disagree) to 5 (strongly agree). Higher scores on the avoidance dimension indicate greater avoidance, whereas higher scores on the other three dimensions indicate greater active preparation. In the present sample, Cronbach’s alpha was 0.921 for the total scale and 0.886, 0.686, 0.901, and 0.852 for the four dimensions, respectively.

Self-support ability was measured using the 45-item Self-supporting Ability Rating Scale for the Elderly (10). Items are rated from 1 (completely inconsistent) to 5 (completely consistent), with total scores ranging from 45 to 225; higher scores indicate better self-support ability. Self-efficacy was assessed using the 10-item Generalized Self-Efficacy Scale (11), rated on a 4-point scale with total scores ranging from 10 to 40; higher scores indicate greater self-efficacy.

### Data collection and quality control

Questionnaires were distributed through village networks using the Wenjuanxing platform. Older adults who could complete the survey independently used the electronic questionnaire, whereas trained investigators provided one-to-one assistance at village sites for participants who were unfamiliar with smartphones or required help reading or entering responses. Investigators received standardized training before data collection. After collection, two researchers independently reviewed questionnaires for completeness and logical consistency. Records with missing key information, evident logical inconsistencies, or identifiable duplicate submissions were excluded. The final analytic dataset contained 851 complete records.

### Statistical analysis

Data were analyzed using SPSS version 25.0 and Mplus version 8.3. Continuous variables were summarized as means and standard deviations, and categorical variables as frequencies and percentages. For univariate comparisons of self-support ability, independent-samples *t* tests were used for variables with two categories and one-way analysis of variance (ANOVA) for variables with three or more categories. Variables showing statistically significant differences in univariate analyses were considered for inclusion in the multiple linear regression model. Self-efficacy and PFC profile membership were also included because of their relevance to the study aims.

LPA was conducted in Mplus using the four PFC dimension mean item scores as continuous indicators, placing all dimensions on a common 1–5 metric. Models containing one to five latent profiles were fitted sequentially. Model fit was evaluated using the Akaike information criterion (AIC), Bayesian information criterion (BIC), adjusted Bayesian information criterion (aBIC), entropy, Lo–Mendell–Rubin likelihood ratio test (LMRT), and bootstrap likelihood ratio test (BLRT) (28). Lower AIC, BIC, and aBIC values indicate better fit, higher entropy indicates greater classification accuracy, and significant LMRT/BLRT results favor a model with k profiles over a model with *k* − 1 profiles. The final solution was selected by jointly considering fit indices, profile size, and substantive interpretability.

After profile classification, multiple linear regression was performed with self-support ability as the dependent variable. Age, marital status, living arrangement, care from children, family members’ health status, number of chronic diseases, monthly household income per capita, self-efficacy, and PFC profile membership were entered in the adjusted model. Categorical predictors were represented by dummy variables; the detailed coding scheme and reference categories are provided in Supplementary Table S3. A two-sided *p* value < 0.05 was considered statistically significant.

## Results

### Sample characteristics and univariate comparisons

The 851 participants included 394 men (46.3%) and 457 women (53.7%). Age was categorized as 60–64, 65–74, 75–84, and ≥85 years. Participant characteristics and univariate comparisons of self-support ability are shown in Table 1. Significant univariate differences in self-support ability were observed according to age, marital status, living arrangement, care from children, family members’ health status, number of chronic diseases, monthly household income per capita, and PFC profile membership (all *p* < 0.05). Gender, educational level, religious belief, and medical accessibility were not significantly associated with self-support ability in univariate comparisons.

**Table 1:** Participant characteristics and univariate comparisons of self-support ability (*n* = 851).

Characteristic	Level	*n* (%)	Self-support ability (M ± SD)	Statistic	*p* value
Gender	Male	394 (46.3)	134.18 ± 48.00	−0.582	0.561
	Female	457 (53.7)	136.09 ± 47.83		
Age	60–64	314 (36.9)	140.16 ± 47.13	3.318	0.019
	65–74	282 (33.1)	135.37 ± 48.55		
	75–84	167 (19.6)	132.08 ± 49.03		
	≥85	88 (10.3)	122.91 ± 44.23		
Marital status	Married	463 (54.4)	138.65 ± 47.95	3.012	0.029
	Widowed	218 (25.6)	134.97 ± 48.18		
	Never married	48 (5.6)	130.04 ± 46.07		
	Divorced	122 (14.3)	124.57 ± 46.67		
Educational level	Illiterate/no formal education	149 (17.5)	136.10 ± 46.93	0.307	0.820
	Primary school	285 (33.5)	136.85 ± 48.92		
	Junior high school	259 (30.4)	133.04 ± 47.46		
	Senior high school or above	158 (18.6)	134.93 ± 47.92		
Religious belief	None	542 (63.7)	137.30 ± 48.15	0.642	0.668
	Buddhism	122 (14.3)	130.97 ± 48.46		
	Taoism	23 (2.7)	135.35 ± 45.39		
	Christianity	119 (14.0)	132.03 ± 48.01		
	Catholicism	21 (2.5)	132.52 ± 53.21		
	Other	24 (2.8)	127.42 ± 35.88		
Living arrangement	Living alone	232 (27.3)	171.91 ± 32.02	91.143	<0.001
	Living with spouse	352 (41.4)	128.03 ± 47.51		
	Living with children	192 (22.6)	116.76 ± 44.34		
	Other	75 (8.8)	102.59 ± 30.60		
Care from children	Very little	13 (1.5)	90.77 ± 38.06	2.964	0.019
	Little	65 (7.6)	134.00 ± 26.28		
	Moderate	243 (28.6)	134.76 ± 38.23		
	High	263 (30.9)	136.38 ± 47.14		
	Very high	267 (31.4)	136.90 ± 58.97		
Family members’ health status	All healthy	329 (38.7)	137.02 ± 48.52	12.629	<0.001
	At least one with chronic disease but independent	372 (43.7)	141.85 ± 46.68		
	At least one requiring long-term care (≥3 months)	30 (3.5)	105.40 ± 37.47		
	Functionally dependent spouse/primary co-resident	120 (14.1)	117.10 ± 45.34		
Number of chronic diseases	0	34 (4.0)	158.68 ± 44.54	5.396	0.005
	1	550 (64.6)	135.95 ± 48.15		
	≥2	267 (31.4)	130.68 ± 46.96		
Monthly household income per capita (CNY)	<1,000	178 (20.9)	131.78 ± 47.92	3.752	0.011
	1,000–1,999	411 (48.3)	132.84 ± 48.31		
	2,000–2,999	218 (25.6)	138.25 ± 47.02		
	≥3,000	44 (5.2)	156.07 ± 43.40		
Medical accessibility (walking time to clinic/village doctor)	<15 min	183 (21.5)	135.92 ± 48.15	1.823	0.141
	15–30 min	444 (52.2)	137.91 ± 47.74		
	31–60 min	174 (20.4)	130.51 ± 47.44		
	>60 min	50 (5.9)	124.88 ± 48.65		
Preparation for future care profile	Scarce-preparation	204 (24.0)	95.06 ± 35.08	278.324	<0.001
	Moderate-preparation	400 (47.0)	129.84 ± 39.20		
	High-avoidance/high-action	247 (29.0)	177.04 ± 35.73		

Independent-samples *t* test was used for variables with two groups; one-way analysis of variance (ANOVA; *F* statistic) was used for variables with three or more groups. M, mean; SD, standard deviation.

### Scores of preparation for future care, self-support ability, and self-efficacy

The mean total PFC score was 42.59 ± 11.47. Mean dimension scores were 9.08 ± 3.27 for avoidance of care planning, 11.98 ± 3.20 for concrete planning, 12.33 ± 4.20 for awareness of future care needs, and 9.19 ± 3.06 for decision-making about care preferences. The mean self-support ability score was 135.20 ± 47.89, and the mean self-efficacy score was 21.25 ± 9.46. Scale- and dimension-level descriptive statistics are presented in Table 2, and item-level descriptive statistics are provided in Supplementary Table S2.

**Table 2:** Descriptive statistics for preparation for future care, self-support ability, and self-efficacy (*n* = 851).

Measure	No. of items	Possible score	Total/dimension score (M ± SD)	Mean item score (M ± SD)
Preparation for future care	14	14–70	42.59 ± 11.47	3.04 ± 0.82
Avoidance of care planning	3	3–15	9.08 ± 3.27	3.03 ± 1.09
Concrete planning	4	4–20	11.98 ± 3.20	3.00 ± 0.80
Awareness of future care needs	4	4–20	12.33 ± 4.20	3.08 ± 1.05
Decision-making about care preferences	3	3–15	9.19 ± 3.06	3.06 ± 1.02
Self-support ability	45	45–225	135.20 ± 47.89	3.00 ± 1.06
Self-efficacy	10	10–40	21.25 ± 9.46	2.13 ± 0.95

Mean item scores are shown on the original item-response metric. PFC and self-support ability items use 1–5 response scales; self-efficacy items use a 1–4 response scale. M, mean; SD, standard deviation; PFC, preparation for future care.

### Model fit and profile selection

As the number of profiles increased, AIC, BIC, and aBIC decreased. The three-profile model showed good classification accuracy (entropy = 0.874), and both the LMRT and BLRT favored the three-profile model over the two-profile model (*p* < 0.001). Although the four-profile model had slightly lower information criteria and higher entropy, one profile contained only 2.35% of the sample, limiting stability and practical interpretability. In the five-profile model, the LMRT was no longer statistically significant (*p* = 0.184). Considering statistical fit, profile size, and interpretability, the three-profile solution was retained. Detailed fit indices are presented in Table 3.

**Table 3:** Model-fit indices for latent profile analysis of preparation for future care.

Model	AIC	BIC	aBIC	Entropy	LMRT (*p*)	BLRT (*p*)	Class proportions (%)
1	9,554.756	9,592.727	9,567.322	–	–	–	100.00
2	8,434.607	8,496.310	8,455.026	0.809	<0.001	<0.001	33.26/66.74
3	7,826.366	7,911.801	7,854.638	0.874	<0.001	<0.001	23.97/47.00/29.03
4	7,738.857	7,848.025	7,774.984	0.897	<0.001	<0.001	24.09/44.54/2.35/29.02
5	7,697.017	7,829.917	7,740.997	0.882	0.184	<0.001	40.66/23.50/5.64/2.70/27.50

AIC, Akaike information criterion; BIC, Bayesian information criterion; aBIC, adjusted Bayesian information criterion; LMRT, Lo–Mendell–Rubin likelihood ratio test; BLRT, bootstrap likelihood ratio test. Lower AIC, BIC, and aBIC values indicate better fit, whereas higher entropy indicates greater classification accuracy. Profile size and substantive interpretability were considered together with statistical fit indices when selecting the final solution.

### Latent profiles of preparation for future care

**Figure 1:**
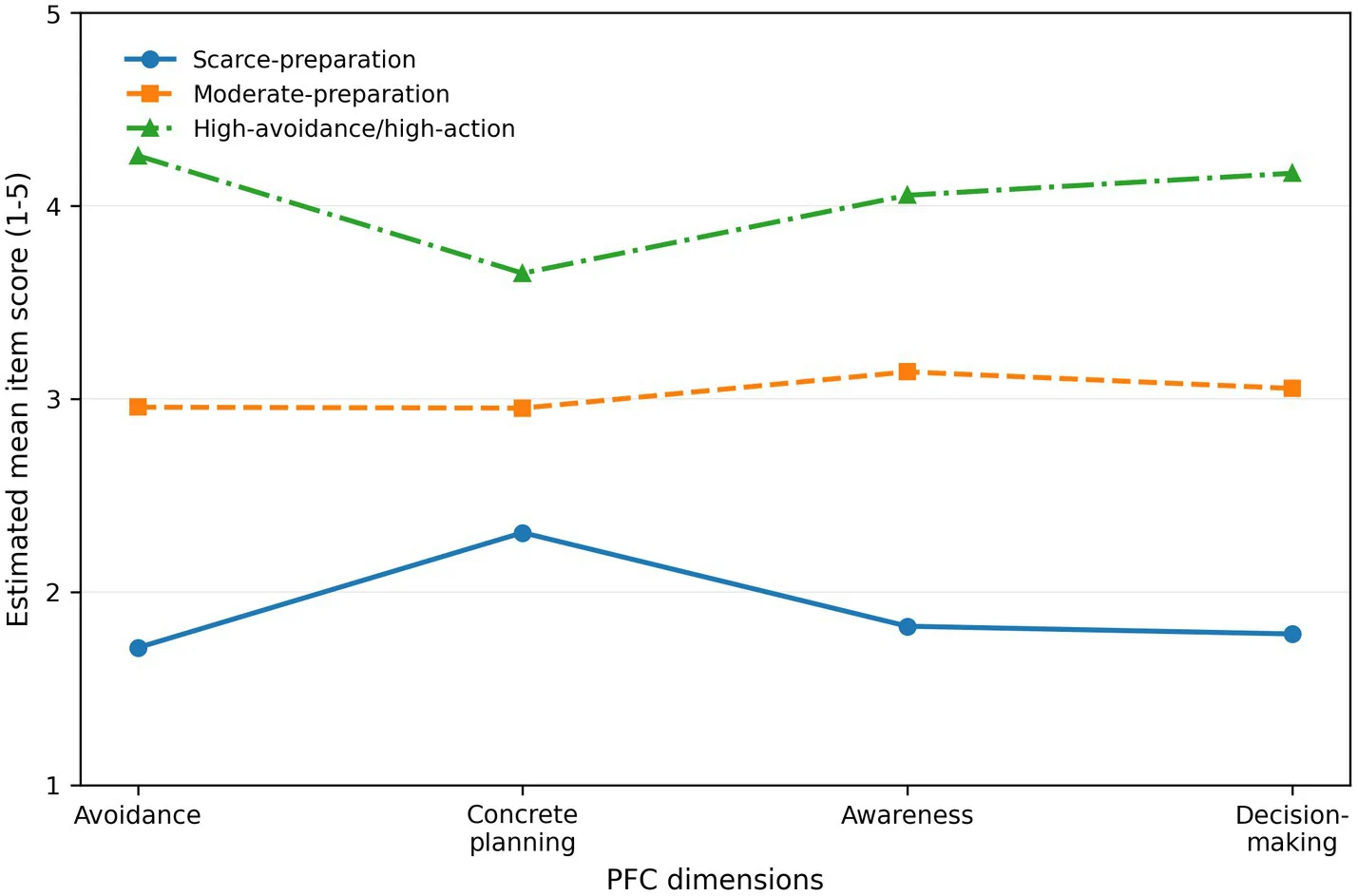
Latent profiles of preparation for future care among rural older adults. The *x*-axis represents the four PFC dimensions used as continuous indicators in the latent profile analysis. The *y*-axis represents the class-specific estimated mean item score on the common 1–5 metric. Higher scores on avoidance indicate greater avoidance of future care planning, whereas higher scores on concrete planning, awareness, and decision-making indicate greater active preparation. Line and marker styles distinguish the three profiles so that interpretation does not depend on color alone.

Three latent profiles were identified. Profile 1, labeled scarce-preparation, included 204 participants (23.97%) and was characterized by low avoidance together with low concrete planning, awareness, and decision-making. Profile 2, labeled moderate-preparation, included 400 participants (47.00%) and showed intermediate levels across all four dimensions. Profile 3, labeled high-avoidance/high-action, included 247 participants (29.03%) and was characterized by high avoidance together with high concrete planning, awareness, and decision-making. Class-specific estimated means are shown in Figure 1.

### Association between PFC profile membership and self-support ability

In the adjusted multiple linear regression model, marital status, family members’ health status, self-efficacy, and PFC profile membership were significant overall predictors of self-support ability. Compared with married participants, widowed participants (*B* = −22.502, *p* < 0.001), never-married participants (*B* = −24.450, *p* < 0.001), and divorced participants (*B* = −25.944, *p* < 0.001) had lower self-support ability. Compared with participants whose family members were all healthy, those with at least one family member requiring long-term care had lower self-support ability (*B* = −20.355, *p* < 0.001), as did those with a functionally dependent spouse or primary co-resident (*B* = −7.372, *p* = 0.028). Self-efficacy was positively associated with self-support ability (*B* = 2.519, *p* < 0.001).

Compared with the high-avoidance/high-action profile, participants in the scarce-preparation profile (*B* = −52.874, *p* < 0.001) and moderate-preparation profile (*B* = −31.296, *p* < 0.001) had substantially lower self-support ability scores. The model explained 64.0% of the variance in self-support ability (*R*^2^ = 0.640, adjusted *R*^2^ = 0.630, *F* = 61.183, *p* < 0.001). Detailed results are shown in Table 4.

**Table 4:** Multiple linear regression of factors associated with self-support ability (*n* = 851).

Predictor	Level	*B*	SE	Standardized *β*	*t*	*p* value
Constant		138.307	10.822		12.780	<0.001
Age	Age 65–74	−1.755	2.431	−0.017	−0.722	0.470
	Age 75–84	−3.683	2.852	−0.031	−1.291	0.197
	Age ≥85	−4.965	3.604	−0.032	−1.378	0.169
Marital status	Widowed	−22.502	3.393	−0.205	−6.632	<0.001
	Never married	−24.450	5.112	−0.118	−4.783	<0.001
	Divorced	−25.944	3.702	−0.190	−7.008	<0.001
Living arrangement	Living with spouse	−3.133	5.015	−0.032	−0.625	0.532
	Living with children	−0.832	4.675	−0.007	−0.178	0.859
	Other living arrangement	−10.095	5.228	−0.060	−1.931	0.054
Care from children	Very little	−21.107	8.558	−0.054	−2.466	0.014
	Little	−6.073	4.240	−0.034	−1.432	0.152
	Moderate	−2.025	2.681	−0.019	−0.756	0.450
	High	−0.656	2.568	−0.006	−0.255	0.798
Family members’ health status	At least one with chronic disease but independent	2.744	2.258	0.028	1.215	0.225
	At least one requiring long-term care (≥3 months)	−20.355	5.705	−0.078	−3.568	<0.001
	Functionally dependent spouse/primary co-resident	−7.372	3.353	−0.054	−2.199	0.028
Number of chronic diseases	1 chronic disease	−6.351	5.497	−0.063	−1.155	0.248
	≥2 chronic diseases	−10.025	5.656	−0.097	−1.773	0.077
Monthly household income per capita (CNY)	<1,000	−3.324	5.084	−0.028	−0.654	0.513
	1,000–1,999	−5.764	4.818	−0.060	−1.196	0.232
	2,000–2,999	−5.254	4.993	−0.048	−1.052	0.293
Preparation for future care profile	Scarce-preparation	−52.874	3.114	−0.472	−16.980	<0.001
	Moderate-preparation	−31.296	2.580	−0.326	−12.129	<0.001
Self-efficacy	Self-efficacy	2.519	0.198	0.497	12.703	<0.001

B, unstandardized regression coefficient; SE, standard error; beta, standardized regression coefficient. Reference categories were age 60–64 years, married, living alone, very high care from children, all family members healthy, no chronic disease, monthly household income ≥3,000 CNY, and the high-avoidance/high-action PFC profile. *R*^2^ = 0.640; adjusted *R*^2^ = 0.630; *F* = 61.183; *p* < 0.001.

## Discussion

This study identified three distinct PFC profiles among rural older adults: scarce-preparation, moderate-preparation, and high-avoidance/high-action. The findings confirm substantial heterogeneity in future care preparation and extend earlier person-centered research among rural Chinese older adults (13). Importantly, the profile pattern shows that avoidance and active preparation can coexist. This finding is consistent with Li et al., who also identified a high-avoidance/high-action subgroup in rural China (13).

The scarce-preparation profile combined low avoidance with low awareness, decision-making, and concrete planning. Thus, low avoidance should not be interpreted as adequate preparation in itself. Participants in the moderate-preparation profile showed intermediate levels across the four dimensions. By contrast, participants in the high-avoidance/high-action profile reported the highest levels of concrete planning, awareness, and decision-making despite also reporting high avoidance. Emotional reluctance to think about possible dependency may therefore coexist with practical efforts to prepare for future care, particularly in sociocultural contexts where future dependency is uncomfortable to discuss but family responsibilities and care needs remain salient. Related evidence on care decision-making and heterogeneous home- and community-care preferences further supports the view that awareness, preferences, and concrete action need not move in parallel (29–31).

The present study should also be distinguished from the CBR mixed-methods protocol reported by Xu et al. (14). That protocol was designed to develop and validate a CBR needs instrument and subsequently conduct a large cross-sectional survey and latent class analysis in Guizhou Province. The present study is not an implementation of any phase of that protocol: it was conducted by an independent research team in Hebei Province, used an established PFC instrument, and examined the association between PFC profiles and self-support ability. The two studies nevertheless provide complementary evidence that care-related needs and preparation are heterogeneous among older adults.

A central finding was the strong association between PFC profile membership and self-support ability. Participants in the scarce-preparation and moderate-preparation profiles had substantially lower self-support ability than those in the high-avoidance/high-action profile after adjustment. This pattern suggests that the action-oriented dimensions of PFC—awareness, decision-making, and concrete planning—may be particularly relevant to later-life autonomy. However, because the study is cross-sectional, the direction of this relationship cannot be determined. Greater self-support ability may facilitate active planning, active preparation may support autonomy, or both may reflect shared personal and family resources.

Self-efficacy was positively associated with self-support ability, consistent with the role of confidence in health management, problem solving, and adaptation to age-related challenges. Marital status was also associated with self-support ability, with widowed, never-married, and divorced participants scoring lower than married participants. Family members’ health status remained relevant after adjustment: having a household member who required long-term care or a functionally dependent spouse/primary co-resident was associated with lower self-support ability. These findings reinforce the importance of family care context and household resource constraints (22, 32–37).

### Practical implications

The findings have several implications for rural primary care and community health management. First, PFC assessment could be incorporated into routine health management alongside chronic disease, functional, and psychosocial assessment. The profile structure suggests that a uniform approach to care-planning education may not adequately address heterogeneous needs. Participants in the scarce-preparation profile should be prioritized because low avoidance did not translate into awareness or concrete action and this group had the lowest self-support ability. Structured education, identification of realistic care options, and stepwise assistance in discussing caregivers and preferred care arrangements may help convert general willingness into specific preparation.

Second, participants in the moderate-preparation profile may benefit from action-oriented support that helps translate intermediate awareness into concrete decisions and arrangements. Those in the high-avoidance/high-action profile already demonstrated substantial planning and decision-making but may still benefit from supportive communication that addresses emotional avoidance and concerns about future dependency. Thus, intervention intensity should be based on the combination of avoidance and active preparation rather than on avoidance alone.

Third, family involvement should be incorporated into profile-based care planning. Practical strategies include structured family discussions about care preferences, clarification of caregiving roles, identification of alternative caregivers if a spouse or adult child becomes unavailable, and early discussion of financial and service arrangements. Village doctors, community nurses, and other primary care professionals may help facilitate these conversations and connect families with available resources. Strengthening the accessibility, continuity, and coordination of community-based services may also make individual care plans more feasible (38–40).

From a broader public health perspective, the specific profile proportions observed in rural Hebei should not be assumed to generalize directly to other countries. Nevertheless, the underlying approach—identifying heterogeneity, stratifying support according to preparation needs, involving families where appropriate, and linking care planning with accessible community services—may be relevant in other rural or resource-constrained settings experiencing rapid population aging. Implementation should be adapted to local primary care infrastructure, cultural expectations, financing arrangements, and availability of formal and informal long-term care.

### Limitations

This study has several limitations. First, the cross-sectional design precludes causal and temporal inference between PFC profile membership and self-support ability. Second, convenience sampling from rural Hebei may limit the representativeness of the sample and the generalizability of profile proportions to other regions. Third, self-reported measures may be affected by recall and social desirability bias. Fourth, site-specific questionnaire distribution counts were not retained in the anonymized analytic dataset, so differential response rates across the 16 villages could not be assessed. Fifth, although demographic, family, and psychosocial variables were included, other potentially relevant factors such as health literacy, detailed caregiving resources, formal long-term care availability, and changes in functional status were not comprehensively examined.

Future research should use longitudinal designs with repeated PFC assessments to determine whether profiles are stable or whether older adults transition between profiles as health, family circumstances, and care resources change. Latent transition analysis could directly examine such changes. Multicenter studies involving geographically and socioeconomically diverse rural regions, preferably with probability-based sampling and documented site-specific recruitment flows, would improve external validity. Future studies should also incorporate objective functional indicators and detailed measures of family and service resources. Intervention studies are needed to test whether profile-tailored care-planning education, family communication strategies, and linkage to community services improve PFC, self-support ability, or other later-life outcomes. Cross-cultural studies could further assess whether comparable PFC profiles occur outside China and how health-system and cultural contexts modify their meaning.

## Conclusion

Three PFC profiles were identified among rural Chinese older adults: scarce-preparation, moderate-preparation, and high-avoidance/high-action. PFC profile membership was strongly associated with self-support ability, with the scarce-preparation and moderate-preparation profiles showing lower adjusted self-support ability than the high-avoidance/high-action profile. These findings demonstrate that low avoidance does not necessarily indicate adequate preparation and that active care-planning behaviors can coexist with avoidance. Profile-informed, family-sensitive, and community-based approaches should be evaluated prospectively before wider implementation.

## Data Availability

The original contributions presented in the study are included in the article/Supplementary material, further inquiries can be directed to the corresponding author.
