# Supplementary material for: Future care preparation profiles and self-support ability among rural Chinese older adults: a cross-sectional study
Source: Front Public Health. 2026 Sep 3;14:1919757. doi: 10.3389/fpubh.2026.1919757 (PMC13581699; doi:10.3389/fpubh.2026.1919757)
Supplement: Supplementary file 1 [file Data_Sheet_1.PDF]

Supplementary Table S1. Rural study sites in Hebei Province

No.	Village	Township/Town	County/District/City	Study-area grouping
1	Xiwangzhuang Village	Xiaojue Town	Pingshan County	Shijiazhuang area
2	Sangjiazhuang Village	Tashang Town	Lingshou County	Shijiazhuang area
3	Xiwan Village	Chenzhuang Town	Lingshou County	Shijiazhuang area
4	Yujia Village	Yujia Township	Jingxing County	Shijiazhuang area
5	Xiaopei Village	Luancheng Town	Luancheng District	Shijiazhuang area
6	Dongliuchun Village	Dongliuchun Township	Dingzhou	Dingzhou area
7	Shaocun Village	Dongliuchun Township	Dingzhou	Dingzhou area
8	Beishaocun Village	Dongliuchun Township	Dingzhou	Dingzhou area
9	Xiwangnou Village	Dongliuchun Township	Dingzhou	Dingzhou area
10	Mafucai Village	Dongliuchun Township	Dingzhou	Dingzhou area
11	Xichaili Village	Dongliuchun Township	Dingzhou	Dingzhou area
12	Lujiazhuang Village	Haotouzhuang Hui Township	Dingzhou	Dingzhou area
13	Wujiazhuang Village	Haotouzhuang Hui Township	Dingzhou	Dingzhou area
14	Majiazhuang Village	Haotouzhuang Hui Township	Dingzhou	Dingzhou area
15	Liuliangzhuang Village	Haotouzhuang Hui Township	Dingzhou	Dingzhou area
16	Wudingzhuang Village	Haotouzhuang Hui Township	Dingzhou	Dingzhou area

Note: The 16 study sites comprised five villages in the Shijiazhuang area and 11 villages in Dingzhou. Site-specific questionnaire distribution counts were not retained in the anonymized analytic dataset; therefore, site-specific response rates could not be reliably reconstructed.

Supplementary Table S2. Item-level descriptive statistics for the PFC, self-support ability, and Generalized Self-Efficacy scales (n = 851)

Scale	Dimension	Item	Mean	SD
Preparation for future care	Avoidance of care planning	Item 5	3.01	1.20
Preparation for future care	Avoidance of care planning	Item 4	3.02	1.20
Preparation for future care	Avoidance of care planning	Item 6	3.05	1.22
Preparation for future care	Concrete planning	Item 14	3.04	1.18
Preparation for future care	Concrete planning	Item 13	3.03	1.19
Preparation for future care	Concrete planning	Item 12	2.90	1.04
Preparation for future care	Concrete planning	Item 9	3.02	1.05
Preparation for future care	Awareness of future care needs	Item 3	3.07	1.19
Preparation for future care	Awareness of future care needs	Item 2	3.07	1.21
Preparation for future care	Awareness of future care needs	Item 1	3.09	1.16
Preparation for future care	Awareness of future care needs	Item 8	3.10	1.23
Preparation for future care	Decision-making about care preferences	Item 10	3.11	1.14
Preparation for future care	Decision-making about care preferences	Item 11	3.08	1.15
Preparation for future care	Decision-making about care preferences	Item 7	3.00	1.20
Self-support ability		Item 1	2.93	1.30
Self-support ability		Item 2	2.90	1.30
Self-support ability		Item 3	2.90	1.28
Self-support ability		Item 4	2.93	1.28
Self-support ability		Item 5	2.99	1.27
Self-support ability		Item 6	2.99	1.31
Self-support ability		Item 7	2.93	1.25
Self-support ability		Item 8	3.00	1.30

Scale	Dimension	Item	Mean	SD
Self-support ability		Item 9	3.00	1.28
Self-support ability		Item 10	3.03	1.35
Self-support ability		Item 11	2.99	1.30
Self-support ability		Item 12	3.03	1.30
Self-support ability		Item 13	3.00	1.29
Self-support ability		Item 14	3.00	1.29
Self-support ability		Item 15	3.06	1.34
Self-support ability		Item 16	2.98	1.31
Self-support ability		Item 17	3.01	1.29
Self-support ability		Item 18	3.03	1.34
Self-support ability		Item 19	2.98	1.28
Self-support ability		Item 20	2.94	1.30
Self-support ability		Item 21	3.02	1.32
Self-support ability		Item 22	3.02	1.32
Self-support ability		Item 23	2.99	1.31
Self-support ability		Item 24	3.01	1.28
Self-support ability		Item 25	2.98	1.30
Self-support ability		Item 26	2.98	1.32
Self-support ability		Item 27	2.96	1.29
Self-support ability		Item 28	3.01	1.33
Self-support ability		Item 29	3.00	1.29
Self-support ability		Item 30	3.00	1.28
Self-support ability		Item 31	3.00	1.29
Self-support ability		Item 32	3.79	0.99
Self-support ability		Item 33	2.94	1.29
Self-support ability		Item 34	2.99	1.30
Self-support ability		Item 35	3.00	1.31
Self-support ability		Item 36	2.98	1.30

Scale	Dimension	Item	Mean	SD
Self-support ability		Item 37	3.01	1.33
Self-support ability		Item 38	3.01	1.27
Self-support ability		Item 39	3.02	1.26
Self-support ability		Item 40	2.95	1.29
Self-support ability		Item 41	2.95	1.31
Self-support ability		Item 42	2.99	1.33
Self-support ability		Item 43	3.02	1.29
Self-support ability		Item 44	2.98	1.32
Self-support ability		Item 45	2.99	1.30
Generalized self-efficacy		Item 1	2.12	1.05
Generalized self-efficacy		Item 2	2.12	1.06
Generalized self-efficacy		Item 3	2.10	1.05
Generalized self-efficacy		Item 4	2.12	1.03
Generalized self-efficacy		Item 5	2.13	1.02
Generalized self-efficacy		Item 6	2.13	1.03
Generalized self-efficacy		Item 7	2.11	1.04
Generalized self-efficacy		Item 8	2.14	1.03
Generalized self-efficacy		Item 9	2.14	1.06
Generalized self-efficacy		Item 10	2.14	1.01

Note: Item numbering follows the original validated scales. PFC, preparation for future care; SD, standard deviation.

Supplementary Table S3. Coding of independent variables in the multiple linear regression model

Variable	Coding
Age	60-64 years = reference; 65-74, 75-84, and >=85 years entered as dummy variables
Marital status	Married = reference; widowed, never married, and divorced entered as dummy variables
Living arrangement	Living alone = reference; living with spouse, living with children, and other entered as dummy variables
Care from children	Very high = reference; very little, little, moderate, and high entered as dummy variables
Family members' health status	All healthy = reference; chronic disease but independent, long-term care requirement, and functionally dependent spouse/primary co-resident entered as dummy variables
Number of chronic diseases	0 = reference; 1 and >=2 entered as dummy variables
Monthly household income per capita	>=3000 CNY = reference; <1000, 1000-1999, and 2000-2999 CNY entered as dummy variables
PFC profile	High-avoidance/high-action = reference; scarce-preparation and moderate-preparation entered as dummy variables
Self-efficacy	Continuous score

Note: Dummy variables were created for categorical predictors. The stated reference category for each variable was coded as 0 for all corresponding dummy variables.
